# Determination of Aflatoxins in Peanut Products in the Northeast Region of São Paulo, Brazil

**DOI:** 10.3390/ijms10010174

**Published:** 2009-01-06

**Authors:** Carlos A. F. Oliveira, Natália B. Gonçalves, Roice E. Rosim, Andrezza M. Fernandes

**Affiliations:** 1 Department of Food Engineering, School of Animal Science and Food Engineering, University of São Paulo, Pirassununga, SP, Brazil. E-Mail: nabiazzi@yahoo.com.br (N. G.); roice@usp.br (R. R.); 2 Department of Microbiology, Institute of Biomedical Sciences, University of São Paulo, São Paulo, SP, Brazil. E-Mail: andrezzaf@hotmail.com

**Keywords:** AFB_1_, mycotoxins, occurrence, peanuts

## Abstract

The aim of the present study was to determine aflatoxin levels in peanut products traded in the Northeast region of São Paulo, Brazil. To this end, 240 samples of peanut products traded in the cities of Araras, Leme, Pirassununga and Porto Ferreira were collected from June 2006 to May 2007. The samples were analyzed for aflatoxins (AF) B_1_, B_2_, G_1_ and G_2_ by high performance liquid chromatography. Results showed 44.2% samples positive for AF at levels of 0.5 to 103.8 μg·kg^−1^. Nine of the positive samples (3.7% of the analysed samples) had total aflatoxin concentrations (B_1_+B_2_+G_1_+G_2_) higher than the limit established by Brazilian regulations (20 μg·kg^−1^). Based on the above data, the probable mean daily intake (PDI_M_) of aflatoxins from peanut products in the Northeast region of São Paulo was estimated to be 0.23 ng kg b.w. day^−1^. Although this PDI_M_ value was relatively low, results indicate that aflatoxin contamination of peanut products may be a public health concern in Brazil, when considering the potential exposure of highly susceptible consumers. For example, it should be emphasized that children are potentially exposed to aflatoxins, since they consume large quantities of peanut candies, and these products had the highest number of samples positive for AFB_1_.

## 1. Introduction

Aflatoxins are secondary metabolites produced by fungi in the genus *Aspergillus*, including *A. flavus*, *A. parasiticus* and *A. nomius* [12]. These fungi grow naturally on food products and the aflatoxins they produce cause a variety of toxic effects in vertebrates, including humans [5]. The tropical climate in Brazil is ideal for the growth and development of these fungi, particularly in peanut products [15].

The aflatoxins identified include B_1_, B_2_, G_1_ and G_2_. Aflatoxin B_1_ (AFB_1_) is most frequently found in plant substrates, and shows the greatest toxigenic potential [12]. Based on previous studies, the IARC (1993) has classified AFB_1_ as a class 1A human carcinogen [16]. In 2002, Brazilian authorities established a maximum limit of 20 μg·kg^−1^ for total aflatoxins (B_1_+B_2_+G_1_+G_2_) in peanuts (unshelled, shelled, raw or roasted), peanut paste (or peanut butter) and corn (whole, crushed, mashed, ground, meals and brans) [1].

The occurrence of aflatoxins in Brazilian foodstuffs has been frequently reported, and is a particular problem in peanuts, with up to 52% of the samples analysed being positive for aflatoxins [15, 18]. Factors responsible for the high incidence of aflatoxin contamination of peanuts include poor agricultural practices during planting, harvesting, drying, transportation and storage of the product. These practices favour fungal contamination and growth, and aflatoxin production [5, 12]. In the state of São Paulo, contamination of unprocessed peanuts and peanut products by AFB_1_ was reported in the period 1988–1999 with mean levels ranging from 51 to 420 μg·kg^−1^ [3, 6–9, 18, 20]. Although the levels of aflatoxins are presumed to be lower in processed foods, due to quality control systems applied by the industry, very little information is available on the occurrence of aflatoxins in Brazilian peanut products directly available for human consumption, especially after the implementation of the tolerance limit in 2002. Therefore, the objective of the present study was to determine aflatoxin concentrations in unprocessed peanuts and in sweet and salty peanut products traded in the Northeast region of the state of São Paulo, Brazil.

## 2. Results and Discussion

Table 1 shows the number of samples of unprocessed peanut, *paçoca*, salty roasted peanut, salty dragee peanut, and sweet dragee peanuts that contained at least one of the aflatoxins B_1_, B_2_, G_1_ or G_2_ at levels equal to or higher than the limit of quantification (0.5 μg·kg^−1^). From the 240 samples analysed, 106 (44.2%) had aflatoxin concentrations ranging from 0.5 to 103.8 μg·kg^−1^. *Paçoca* was the product that had the greatest number of positive samples (72.9%) with a mean aflatoxin level (B_1_+B_2_+G_1_+G_2_) of 8.97 ± 1.61 μg·kg^−1^. Unprocessed peanuts had the highest mean concentration of total aflatoxins (12.88 ± 2.42 μg·kg^−1^) and this occurred in 39.6% of samples analyzed.

The number and percent of samples of each type of product showing levels above the maximum limit for total aflatoxins (20 μg·kg^−1^) as determined by Brazilian regulations [1] are summarized in Table 1. Nine samples (3.7% of the total analyzed) had levels above 20 μg·kg^−1^, with a mean total aflatoxin concentration (B_1_+B_2_+G_1_+G_2_) equal to 50.51 μg·kg^−1^. These samples included 4 samples of *paçoca*, 4 samples of unprocessed peanut, and 1 sample of salty dragee peanut that had mean concentrations of total aflatoxins of 55.52 μg·kg^−1^, 55.72 μg·kg^−1^and 40.31 μg·kg^−1^, respectively. No sample of salty roasted or sweet dragee had aflatoxin concentrations above the tolerance limit adopted in Brazil.

The incidence of aflatoxin in peanut products observed in the present study is lower than those reported by other authors for peanut and peanut products surveyed from 1988–1999 in cities in the state of São Paulo [3, 6–9, 18, 20]. In Campinas, of 66 samples evaluated in 1994, 47% had levels higher than 20 μg·kg^−1^, with some samples reaching up to 997 μg·kg^−1^ [3]. The high incidence of aflatoxins in Campinas was confirmed in 1995 and 1996, when 51% of samples of peanuts and peanut products had aflatoxin concentrations ranging from 43 to 1,099 μg·kg^−1^ [9]. In São José do Rio Preto, Santos *et al*. [20], observed aflatoxin contamination in 39.3% of 178 peanut product samples. In São Paulo, of 1,374 samples evaluated, 68.7% had aflatoxin at levels above 20 μg·kg^−1^ [18]. Higher numbers of samples containing levels above the tolerance limit of 20 μg·kg^−1^ were also observed in cities of other Brazilian states, such as Recife and Goiânia, in surveys conducted in the 1990’s [4, 13]. Compared with those previous reports, results of our trial indicate that, even though 3.7% of the analysed samples showed levels above 20 μg·kg^−1^, the companies that manufacture peanut products have markedly improved the quality control practices for aflatoxins in the state of São Paulo. In Brazil, good manufacturing practices (GMP) have become compulsory for peanut industries since 2003, when the National Health Surveillance Agency issued the regulation [2]. Therefore, the decrease in aflatoxin in Brazilian peanut products in recent years may be a consequence of specific changes in industries that have implemented GMP.

In the present study, *paçoca* and unprocessed peanut had the highest incidence of aflatoxin contamination, which is consistent with data reported earlier in surveys showing higher levels of aflatoxins in those products collected in other regions of the state of São Paulo [7, 8, 14, 19]. *Paçoca* is a traditional ground candy which is mostly consumed by children in Brazil, therefore the consumption of these products contaminated with aflatoxins may pose a significant risk to human health.

The incidence of aflatoxin in peanut products found in our study was in general lower than previous reports conducted in other countries, especially Asia and Africa. Rustom [17] summarized the data on aflatoxin contamination in peanuts and peanut products from several countries during 1982–1994, including Senegal, Mexico, United States, Philippines, India, UK and Nigeria, and concluded that aflatoxin occurrence is extremely variable worldwide, with incidences ranging from 30 to 100%, at levels up to 2,888 μg·kg^−1^. In a recent report, Yentür *et al*. [24] analysed 20 samples of peanut butter from one company in Turkey, and found that all samples contained aflatoxin with total aflatoxins (B_1_+B_2_+G_1_) ranging from 8.16 to 75.74 μg·kg^−1^. However, a comparison between the results of the present study and those reported by other authors is difficult, because the contamination of foods varies according to the area, climatic differences, and agricultural practices, among other factors [22].

One of the most important aspects of risk analysis for chemical substances is the determination of the level of human exposure [23]. In relation to contaminants found in foodstuffs, this determination is particularly difficult, although it may be estimated indirectly based on data on the consumption of contaminated food, and on the respective mean occurrence of the substance. In these conditions, the level of exposure is measured as a function of mean daily intake per body weight unit, and is normally expressed in ng·kg^−1^ of body weight (b.w.) day or week^−1^ [23].

Considering the mean levels of total aflatoxins obtained in the present study, as well as the estimate of peanut product consumption, the probable daily intake of aflatoxins was calculated. Annual *per capita* consumption of peanut products (classified as confectionery products) as estimated in 2004 by the Instituto Brasileiro de Geografia e Estatística [10] for the state of São Paulo is 693 g·person^−1^, which comprises 1.9 g person·day^−1^. Using this value together with the mean concentration of aflatoxins in positive samples of all products analysed (6.05 μg·kg^−1^), the probable daily mean intake (PDI_M_) of aflatoxins would be 11.5 ng (6.05 ng·g–1 × 1.9 g), coming to a PDI_M_ equal to 0.23 ng kg b.w.day^−1^ (considering individuals of 50 kg mean weight). When using the highest mean aflatoxin level obtained in unprocessed peanut (12.88 μg·kg^−1^), the worst case of aflatoxin ingestion would be 24.5 ng, leading to a PDI_M_ of 0.49 ng kg b.w. day^−1^.

The calculated ingestion values indicate that mean aflatoxin levels found in samples of peanut and peanut products in the Northeast region of São Paulo may reach PDI_M_ values close to the mean aflatoxin ingestion estimated in countries with relatively lower incidence of aflatoxins when compared to Brazil, such as the United States (0.26 ng kg b.w. day^−1^) or the European Union (0.47 ng kg b.w. day^−1^) [23]. However, it should be emphasized that AFB_1_ is a potent genotoxic substance, and that peanut products, mainly sweet ones, such as *paçoca* are largely consumed by children. This fact may contribute to an even greater increase in the human health risk posed by the ingestion of aflatoxins in foodstuffs. It is well recognized that aflatoxins cause liver cancer and have additional important toxic effects in farm and laboratory animals, such as interference in immunity, protein metabolism and multiple micronutrients [5]. Although these effects have not been widely studied in humans, the available information indicates that changes in nutrition and immunity may occur in human populations chronically exposed to the aflatoxins through the diet [22].

## 3. Experimental Section

### 3.1. Sampling

Samples were collected in the cities of Araras, Leme, Pirassununga and Porto Ferreira, all located in the Northeast of the state of São Paulo. In each city, samples from five types of peanut products from the brands that showed the greatest trade volume in supermarkets were collected: unprocessed peanut (shelled), roasted and salted peanut (peeled), salty dragee peanut, sweet dragee peanut (confectioneries) and *paçoca* (a traditional Brazilian ground peanut candy). Each month, four samples of each product were collected, making up a total of 20 samples per month. Throughout the 12-month study, 48 samples of each type of product were analysed, for a total of 240 samples.

The sampling unit was made up of original closed packages of at least 500 g, collected from different batches as indicated on the label. Samples were identified, including data on the manufacturer, batch and/or manufacturing date, and expiration date. Products were stored in their respective packages and kept at room temperature (similar to conditions in the supermarkets) until the moment of analysis.

### 3.2. Aflatoxin determination

Analysis of aflatoxins was performed using immunoaffinity columns, as described below. Identification and determination of aflatoxins B_1_, B_2_, G_1_ and G_2_ in peanut product samples were carried out by high performance liquid chromatography according to the method recommended by Scott [21] (AOAC method 980.20 item I), with some modifications.

All samples were ground and homogenized in a hammer mill (~14 mesh). Samples of raw ground peanuts were mixed with distilled water 1:1 and blended. An aliquot of the sample (25g or 30 g for the mixture) was placed in an Erlenmeyer flask containing NaCl (5 g) and methanol/water (125 mL, 70:30, v/v). Samples were placed in an orbital shaker (Tecnal, Piracicaba, Brazil) for 30 minutes, filtered, and an aliquot (15 mL) was transferred to a 50 mL beaker. Thirty mL of ultra pure water (Milli Q, Millipore, Billerica, MA, USA) were added to the extract, and the homogenized mixture was filtered through a 1.5 μm microfibre filter. An aliquot (15 mL) was then passed through the immunoaffinity column (Vicam, Watertown, MA, USA). After washing with ultra pure water (10 mL) the column was eluted with methanol (1 mL) and the eluate was collected in an amber vial. Solvent flow in the columns was kept at 2–3 mL·min^−1^.

The eluate was evaporated to dryness under N_2_. Derivatisation of AFB_1_ and AFG_1_ was obtained by adding trifluoroacetic acid (TFA, 100 μL) and n-hexane (200 μL). The mixture was kept at 35°C for 10 min, then evaporated to near-dryness and diluted in methanol/water/acetonitrile (500 μL, 60:20:20, v/v/v).

Final extracts were filtered through a 0.45 μm PTFE membrane and 20 μL were injected into a high performance liquid chromatography column, using a Shimadzu 10VP liquid chromatograph (Kyoto, Japan) with a 10 AXL fluorescence detector (excitation at 360 nm and emission above 440 nm). A Phenomenex (Torrance, CA, USA) C_18_ column (4.6 × 150 mm, 4 μm) and a Shim-Pack pre-column (4 × 10 mm, 5 μm CLC G-ODS) were used. The isocratic mobile phase consisted of methanol/water/acetonitrile (60:20:20, v/v/v) with a flow rate of 1.0 mL·min^−1^.

Calibration curves were prepared using standard solutions of aflatoxins B_1_, B_2_, G_1_ and G_2_ (Sigma, St Louis, MO, USA) previously evaluated individually according to Scott [20] (AOAC method 971.22). Individual aflatoxin solutions were mixed in convenient volumes to produce working solutions of 3.05 ng·mL^−1^, 6.10 ng·mL^−1^, 12.25 ng·mL^−1^ and 24.50 ng·mL^−1^ of each aflatoxin. As with sample extracts, working solutions were prepared with TFA.

**Figure 1.**
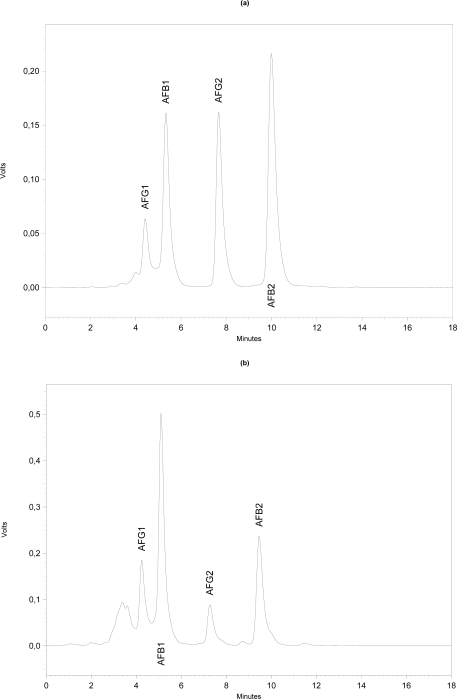
**(a)** Chromatogram showing the retention times of standards of aflatoxins B_1_ (derivatised), B_2_, G_1_ (derivatised) and G_2_ (nearly 5.39, 10.09, 4.45 and 7.62 min, respectively). **(b)** Chromatogram of aflatoxins in naturally contaminated peanut candy (paçoca) containing AFB_1_ (19,90 μg**·**kg^−1^), AFB_2_ (11.43 μg**·**kg^−1^), AFG_1_ (7.06 μg**·**kg^−1^) and AFG_2_ (3.30 μg**·**kg^−1^).

Aflatoxins B_1_ (derivatised), B_2_, G_1_ (derivatised) and G_2_ had retention times of approximately 5.39, 10.09, 4.45 and 7.62 minutes, respectively (Figure 1a). Figure 1b shows a typical chromatogram of a sample of *paçoca* containing all the aflatoxins. Quantification limits for the aflatoxins (B_1_, B_2_, G_1_, G_2_) was 0.5 μg·kg^−1^ for each toxin, as determined by the minimum amount of toxin that could generate a chromatographic peak three times over the baseline standard deviation. The mean coefficient of determination (r) values obtained for AFB_1_, AFB_2_, AFG_1_ and AFG_2_ standard curves were 0.992, 0.943, 0.995 and 0.991, respectively.

Mean recovery percentage of aflatoxins in the validation assay for *paçoca*, unprocessed peanut, salty dragee peanut, sweet dragee peanut and salty roasted peanut spiked with 2 and 20 μg·AF·kg^−1^ are presented in Table 2.

## 4. Conclusions

Samples of peanut products traded in the Northeast region of the state of São Paulo had high levels of aflatoxins, although only 3.7% showed concentrations above the tolerance limit established by Brazilian regulations. The highest aflatoxin levels were observed in samples of ground peanut candy (*paçoca*), unprocessed peanut, and salty dragee peanut. Peanut candies showed the highest number of samples positive for AFB_1_, and this fact warrants concern when considering that children are high consumers of those products. Although the incidence of aflatoxins in peanut products has decreased and the estimated PDI_M_ values were relatively low, results indicate that aflatoxin contamination of peanut products may be a public health concern in Brazil, since it contributes to the general human exposure to these toxins, especially among children.

## Figures and Tables

**Table 1.** Aflatoxin levels in peanut products traded in the Northeast region of the state of São Paulo, Brazil.

Peanut product	Samples with aflatoxin^a^		Aflatoxin level^b^(μg·kg^−1^)				
	>0.5 μg·kg^−1^^c^N (%)	> 20 μg·kg^−1^^d^N (%)	B_1_	B_2_	G_1_	G_2_	Total aflatoxins^e^
Unprocessed	19 (39.6%)	4 (8.3%)	6.02 ± 13.58	0.74 ± 1.34	4.39 ± 10.30	1.73 ± 4.13	12.88 ± 2.42
*Paçoca*^f^	35 (72.9%)	4 (8.3%)	4.33 ± 8.58	1.30 ± 2.88	2.63 ± 6.99	0.70 ± 1.97	8.97 ± 1.61
Salty roasted	15 (31.2%)	0	0.61 ± 0.76	<LOQ^c^	0.55 ± 0.77	<LOQ	1.60 ± 0.25
Salty dragee	20 (41.7%)	1 (2.1%)	1.78 ± 7.32	<LOQ	0.83 ± 1.19	<LOQ	3.32 ± 0.67
Sweet dragee	17 (35.4%)	0	1.47 ± 3.56	<LOQ	0.90 ± 0.68	0.73 ± 1.74	3.47 ± 0.46
Total	106 (44.2%)	9 (3.7%)	2.84 ± 2.26	0.56 ± 0.48	1.86 ± 1.64	0.79 ± 0.55	6.05 ± 4.72

a Number of samples analyzed: 48 for each peanut product (total: 240 samples).

b Results expressed as mean ± standard deviation of samples analyzed in duplicate.

c Limit of quantification (LOQ) of the analytical method (0.5 μg·kg^−1^ for each aflatoxin).

d Tolerance limit adopted in Brazil (sum of aflatoxins B_1_, B_2_, G_1_ and G_2_). ^e^ Sum of aflatoxins B_1_, B_2_, G_1_ and G_2_.

f Ground candy.

**Table 2.** Recoveries of aflatoxins in spiked samples of peanut products.

Product	Spiking level (μg·kg^−1^)	Recovery^a^(%)	RSD^b^(%)
*Paçoca*^c^	2	72.0	4.3
	20	77.2	25.0
Unprocessed peanut	2	108.6	11.8
	20	70.3	9.3
Salty dragee peanut	2	98.8	35.8
	20	71.5	1.8
Sweet dragee peanut	2	92.4	9.9
	20	76.1	14.0
Salty roasted peanut	2	126.5	14.9
	20	91.8	38.8

a Values are reported as means of five replicates.

b Relative standard deviation.

c Ground candy.

## References

[1] Agência Nacional de Vigilância Sanitária Resolução RDC nº 274, de 15 de outubro de 2002 Available at http://e-legis.bvs.br/leisref/public/showAct.php?id=1653&word=Aflatoxina%20M1, acessed September 15, 2008.

[2] Agência Nacional de Vigilância Sanitária Resolução RDC nº 172, de 4 de julho de 2003 Available at http://e-legis.anvisa.gov.br/leisref/public/showAct.php?id=7948, acessed November 03, 2008.

[3] Brigido BM, Badolato MIC, Freitas VPS (1995). Contaminaçäo de amendoim e seus produtos comercializados na regiäo de Campinas-SP, por aflatoxinas durante o ano de 1994. Rev. Inst. Adolfo Lutz..

[4] Colaço W, Ferraz U, Albuquerque LR (1994). Incidência de aflatoxinas em amendoim e produtos derivados consumidos na cidade de Recife, no período de 1989 a 1991. *Rev. Inst.*. Adolfo Lutz.

[5] Coulombe RA, Sharma RP, Salunkhe DK (1991). Aflatoxins. Mycotoxins and phytoalexins.

[6] Fonseca H, Valarini I, Domingues MAC, Wettstein ASR, Silva AEG (1991). Ocorrência de aflatoxina em amendoim, no Estado de São Paulo, durante os anos de 1988–1989. Anais Escola Superior de Agricultura Luiz de Queiroz.

[7] Fonseca H, Calori-Domingues MA, Glória EM, Zambello IV, Segatti-Piedade F (1998). Ocorrência de amendoim contaminado no Estado de São Paulo nos anos de 1990 a 1996. Encontro Nacional de Aflatoxinas.

[8] Freitas VPS, Badolato MIC (1992). Incidência de aflatoxinas em paçocas de amendoim consumidas na cidade de Campinas, estado de São Paulo. Rev Inst Adolfo Lutz.

[9] Freitas VPS, Brigido BM (1998). Occurrence of aflatoxins B_1_, B_2_, G_1_ and G_2_ in peanuts and their products marketed in the region of Campinas, Brazil in 1995 and 1996. Food Addit. Contam..

[10] Instituto Brasileiro de Geografia e Estatística (2004). Pesquisa de orçamentos familiares 2003–2004.

[11] International Agency for Research on Cancer (1993). Some Naturally Occurring Substances: Food Items and Constituents, Heterocyclic Aromatic Amines and Mycotoxins. Monographs on the Evaluation of Carcinogenic Risks to Humans.

[12] Moss MO (1998). Recent studies of mycotoxins. J. Appl. Microbiol-Symposium Suppl.

[13] Oliveira V, Mesquita AJ, Serafini AB, Ribeiro JL, Silva MRR (1991). Ocorrência de aflatoxinas B_1_ e G_1_, em amendoim comercializado em Goiânia - GO, Brasil. Rev. Microbiol.

[14] Ricciardi JA, Ferreira JF (1993). Dosagem de aflatoxina AFB1 em amendoim e doces de amendoim. Rev. Farmácia Bioquímica.

[15] Rodriguez-Amaya DB, Sabino M (2002). Mycotoxin research in Brazil: The last decade in review. Brazilian. J. Microbiol.

[16] Rothschild LJ (1992). IARC classes AFB_1_ as class 1 human carcinogen. Food Chem. News.

[17] Rustom IYS (1997). Aflatoxin in food and feed: Occurrence, legislation and inactivation by physical methods. Food Chem.

[18] Sabino M, Zorzetto AP, Pedroso MO, Milanez TV (1989). Incidência de aflatoxinas em amendoim e produtos derivados consumidos na cidade de São Paulo, no período de 1980 a 1987. Rev. Inst. Adolfo Lutz.

[19] Sabino M, Lamardo LCA, Milanes TV, Inomata EI, Zorzetto MAP, Navas SA, Galvão MS (2000). Twenty years of Aflatoxin contamination in groundnuts and groundnuts products in São Paulo State, Brazil. International IUPAC symposium on mycotoxins and phycotoxins.

[20] Santos CCM, Lopes MRV, Kosseki SY (2001). Ocorrência de aflatoxinas em amendoim e produtos de amendoim comercializados na região de São José do Rio Preto/SP. Rev. Inst. Adolfo Lutz.

[21] Scott PM, Helrich K (1990). Natural poisons. Official methods of analysis of the Association of Official Analytical Chemists.

[22] Williams JH, Phillips TD, Jolly PE, Stiles JK, Jolly CM, Aggarwal D (2004). Human aflatoxicosis in developing countries: A review of toxicology, exposure, potential health consequences, and interventions. Am. J. Cl. Nutr.

[23] World Health Organization (1998). Safety evaluation of certain food additives and mycotoxins. WHO Food Additive Series.

[24] Yentür G, Er B, Özkan MG, Öktem AB (2006). Determination of aflatoxins in peanut butter and sesame samples using high-performance liquid chromatography method. Eur. Food Res. Technol.

